# Effect of FDG PET-CT for Staging and Radiotherapy Planning – A Comparison of Cohorts From Two Randomized Trials of Thoracic Radiotherapy in Limited-Stage SCLC

**DOI:** 10.1016/j.jtocrr.2024.100688

**Published:** 2024-05-16

**Authors:** Gustav Graabak, Bjørn Henning Grønberg, Kristin Toftaker Killingberg, Tarje Onsøien Halvorsen

**Affiliations:** a Department of Clinical and Molecular Medicine, NTNU, Norwegian University of Science and Technology, Trondheim, Norway; b Department of Oncology, St Olav’s Hospital, Trondheim University Hospital, Trondheim, Norway

**Keywords:** LS SCLC, PET-CT, Target volume, Thoracic radiotherapy, Survival, toxicity

## Abstract

**Introduction:**

^18^F-fluorodeoxyglucose positron emission tomography-computed tomography (PET-CT) is recommended for staging and defining target volume in limited-stage SCLC, though the impact on outcomes compared with CT staging and elective nodal irradiation (ENI) is not well documented. We analyzed patients receiving 45 Gy/30 fractions in two randomized trials of thoracic radiotherapy (TRT) in limited-stage SCLC (HAST and THORA trials) to evaluate whether PET-CT for staging and radiotherapy planning reduces radiotoxicity and improves survival.

**Methods:**

Patients in HAST were staged with CT of the thorax and upper abdomen and brain magnetic resonance imaging of the brain. Patients in THORA were staged with PET-CT in addition. All patients were to receive four courses of platinum/etoposide chemotherapy and concurrent TRT starting three to four weeks after the first chemotherapy course. In HAST, target volumes included pathological lesions on CT plus ENI of lymph node stations 4–7 (bilateral). In THORA, target volumes were limited to PET-CT-positive lesions (selective nodal irradiation [SNI]).

**Results:**

A total of 149 patients were included (PET-CT/SNI: n = 76, CT/ENI: n=73); the median age was 64 years, 56% were women, 85% had PS 0 to 1, and 81% had stage III disease. The PET-CT/SNI group experienced less grade 3-4 esophagitis (18% versus 33%, *p* = 0.043), less grade >=1 pneumonitis (5% versus 16%, *p* = 0.028), and less dysphagia after TRT (mean scores on European Organisation for Research and Treatment of Cancer 13-item lung cancer module: 45 versus 72). There was no difference in median overall survival (24 versus 25 mo, *p* = 0.59) or progression-free survival (11 versus 11 mo, *p* = 0.23).

**Conclusions:**

Using PET-CT for staging and target volume definition of TRT reduces acute radiotoxicity but does not improve overall or progression-free survival in limited-stage SCLC.

## Introduction

SCLC is the most aggressive type of lung cancer and accounts for 13% to 15% of all cases.^1^^,^^2^ Platinum-etoposide chemotherapy and concurrent thoracic radiotherapy (TRT) is the standard treatment if all lesions can be included in a radiotherapy field (“limited-stage”, LS),^3^, ^4^, ^5^ and up to 40% of patients are alive five years after chemoradiotherapy.^6^, ^7^, ^8^

A contrast-enhanced computed tomography (CT) scan of the thorax and upper abdomen and magnetic resonance imaging (MRI) of the brain, supplemented with bone scintigraphy when bone metastases were suspected, used to be standard staging modalities of SCLC. ^18^F-fluorodeoxyglucose positron emission tomography-computed tomography (PET-CT) (PET-CT) is more accurate in the assessment of disease extent and separation between LS and extensive stage than CT,^9^, ^10^, ^11^, ^12^, ^13^, ^14^ and studies suggest that elective nodal irradiation (ENI) can be omitted when limiting target volumes to PET-CT positive lesions since less than 3% of these patients experience isolated mediastinal nodal failure.^15^, ^16^, ^17^, ^18^, ^19^ Omission of ENI reduces the irradiated volume and should thereby reduce radiotoxicity, which has been the main limitation of the use of TRT (especially twice-daily TRT) in LS SCLC.^20^^,^^21^ Thus, guidelines recommend using PET-CT for staging and definition of selective nodal irradiation (SNI) in LS SCLC,^22^, ^23^, ^24^, ^25^, ^26^ and PET-CT is increasingly used in clinical practice.^2^^,^^27^

There is, however, limited evidence on whether using PET-CT improves outcomes in SCLC since this has not been investigated in any prospective, randomized trial. A few retrospective studies suggest that using PET-CT improves survival,^27^^,^^28^ while there was no significant difference in survival or acute radiotoxicity between patients staged with (57%) and without PET-CT in the phase III CONVERT trial.^29^

Our group has conducted two randomized phase II trials comparing TRT schedules in LS SCLC (HAST: twice-daily 45 Gy/30 fractions versus once-daily 42 Gy/15 fractions and “THORA”: twice-daily 60 Gy/40 fractions versus 45 Gy/30 fractions).^30^^,^^31^ In HAST, patients were staged with CT and received ENI,^30^ in THORA, all patients underwent a PET-CT for staging and received SNI.^31^ The aim of the present study was to compare survival and radiotoxicity between patients who received twice-daily TRT of 45 Gy/30 fractions in these trials to provide more data on the potential clinical impact of PET-CT for staging and target volume definition in LS SCLC.

## Material and Methods

### Enrollment and Approvals

The HAST trial enrolled patients at 18 hospitals in Norway from May 2005 until January 2011. The THORA trial (NCT02041845) enrolled patients at 22 hospitals in Norway, Sweden, and Denmark from July 2014 until June 2018. Both trials were approved by regulatory authorities in participating countries.^30^^,^^31^

### Eligibility Criteria and Diagnostic Workup

In both trials, eligible patients had confirmed, inoperable SCLC confined to one hemithorax, the mediastinum, contralateral hilus, and supraclavicular regions^32^; were greater than or equal to 18 years old; had Eastern Cooperative Oncology Group (ECOG) performance status of 0 to 2; adequate organ functions; no malignant cells in pleural fluid; no other active cancer; were treatment naïve; and gave written informed consent. Details are presented in Supplementary Table 1.

In HAST, patients were staged with a CT thorax/upper abdomen, brain MRI, and bone scintigraphy. In THORA, all patients underwent a whole-body PET-CT and brain MRI.

### Treatment

In both cohorts, patients were to receive four courses of cisplatin (75 mg/m^2^) or carboplatin (area under the curve of 5–6 mL × min, Calvert’s formula) on day 1 and etoposide (100 mg/m^2^ iv) on days 1–3 every three weeks.

Radiotherapy procedures are listed in Supplementary Table 2. Briefly, TRT commenced 21 to 28 days after the first day of the first chemotherapy course. In HAST, the target volume included all pathological lesions visible on the CT scan and ENI of lymph node stations 4 to 7 (bilateral) with margins (CT/ENI group). In THORA, ENI was omitted, and the target volume was limited to only include PET-CT-positive lesions (PET-CT/SNI group).

There were some differences in normal tissue constraints and clinical and internal target volume (ITV) margins (Supplementary Table 2). Most importantly, less than 50% of the normal lung tissue was to receive 20 Gy or more in the CT/ENI group, while less than 35% of the normal lung tissue was to receive 20 Gy or more, and less than 65% was to receive 5 Gy or more in the PET-CT/SNI group. In both cohorts, the gross tumor volume was delineated on a planning CT scan performed after the first course of chemotherapy. A four-dimensional CT scan for ITV definition was allowed for the PET-CT/SNI group (unavailable for the CT/ENI group). Setup margins for planning target volumes (PTV) were defined according to local routines at each radiotherapy department. For this study, a 5 mm margin was added to the ITV in all directions if PTV was not reported.

Three-dimensional conformal radiotherapy (3D CRT) was the minimum required radiotherapy technique. Intensity-modulated radiotherapy (IMRT) and volumetric-modulated arc therapy (VMAT) were allowed for the PET-CT/SNI group (unavailable for the CT/ENI group). Patients received two fractions per day five days per week with a minimum of six hours between fractions.

Responders to chemoradiotherapy were offered prophylactic cranial irradiation (PCI) of 30 Gy/15 fractions or 25 Gy/10 fractions, starting within six weeks after the first day of the last chemotherapy course.^30^^,^^31^

### Patient Selection

**Figure 1:**
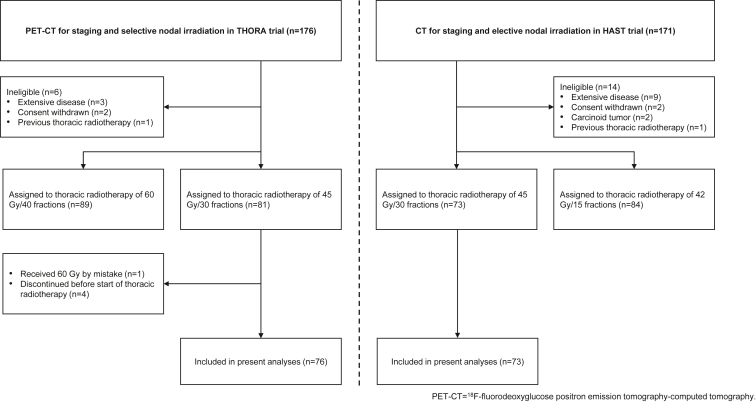
Patient selection. CT, computed tomography; HAST, Higher Ability Selection Test; PET, positron emission tomography.

Patients who were randomly assigned to and commenced twice-daily TRT of 45 Gy/30 fractions in the two trials were included in the present study (Fig. 1).

### Assessments

The stage of disease was assessed according to TNM version 7, treatment response according to Response Evaluation Criteria in Solid Tumours version 1.0 (CT/ENI group) and version 1.1 (PET-CT/SNI group)^33^ on a CT scan within three weeks after completion of chemoradiotherapy. The most important difference between version 1.0 and version 1.1 in this setting is the definition of a pathologically enlarged lymph node (version 1.0: ³10 mm in longest diameter, version 1.1: ³15 mm in short axis).^34^^,^^35^

Toxicity was assessed according to Common Terminology Criteria for Adverse Events version 3.0 (CT/ENI group) and version 4.0 (PET-CT/SNI group).^36^ There are no relevant differences between these versions in definitions of esophagitis and pneumonitis. Patients reported health-related quality of life (HRQoL) on the European Organization for Research and Treatment of Cancer Quality of Life Questionnaire-Core 30 version 3 and its lung cancer-specific module. Questionnaires completed at week 0 (baseline), 3 (within 1 week before TRT), 7 (within 1 week after TRT), 12 (response evaluation), 18 (within 1 week after PCI), and 52 were compared in the present study.

Data collection was completed in March 2022 for the CT/ENI group and in September 2023 for the PET-CT/SNI group (median follow-up was 166 and 92 mo for overall survival [OS], 56 and 64 mo for progression-free survival [PFS] in the CT/ENI and PET-CT/SNI groups, respectively).

### End Points

The primary endpoint was OS, defined as the time from initiation of chemotherapy until death from any cause. Secondary endpoints were 5-year survival rate, PFS (defined as the time from initiation of chemotherapy until disease progression or death from any cause), frequencies and severity of esophagitis and pneumonitis, and HRQoL (dysphagia/LC13, dyspnea/LC13, global QoL/C30).

### Statistical Considerations

Raw scores from the QLQs were converted to a scale from 0 to 100 using the European Organization for Research and Treatment of Cancer scoring manual.^37^ A difference in mean score of 10 or more was considered clinically significant.^38^

OS and PFS were estimated using the Kaplan-Meier method and compared using the Cox proportional hazard method. A Cox model and logistic regression were used for multivariable analyses of OS and 5-year OS, respectively, after all patients had been followed until death or a minimum of five years. Both models were adjusted for baseline characteristics (age [continuous], sex, ECOG performance status, and disease stage). Patients with missing values were excluded from the multivariable analyses. For group comparison, the Pearson’s Chi-square test and Fisher Exact test were used for proportions, the independent samples *t*-test was used for normally distributed data (age), while the Wilcoxon rank-sum test was used for nonparametric data (mean chemotherapy courses, PTV). A two-sided *p*-value of 0.05 or less was considered statistically significant. Analyses were performed using SPSS Statistics (IBM) version 29.

## Results

### Patients

Overall, 154 eligible patients were randomly assigned to TRT of 45 Gy/30 fractions in the two trials. We excluded one patient who received 60 Gy by mistake and four who did not commence TRT in the PET-CT/SNI group. Thus, 149 were included in the present analyses (PET-CT/SNI: 76 [51%]), CT/ENI: 73 [49%]) (Fig. 1).

Median age was 64 years (range: 36-80), 83 patients (56%) were women, 127 patients (85%) had ECOG performance status 0 to 1, 121 patients (81%) stage III disease, 12 patients (8%) pleural fluid, and 39 patients (26%) weight loss greater than or equal to 5% the last three months before inclusion. Overall, the disease stage was similar, though the PET-CT/SNI group had a lower T stage and higher N stage compared with the CT/ENI group. Numerically, the proportion with ECOG performance status 0 was higher in the PET-CT/SNI group (45% versus 27%, *p* = 0.062). Other baseline characteristics were balanced between the two groups (Table 1).

**Table 1:** Baseline Characteristics

Variable	xx	PET-CT/SNI group (n=76)	CT/ENI group (n=73)	p
Age	Median (range)	65 (36–80)	63 (44–79)	0.098
	≥70 years	25 (33%)	17 (23%)	0.19
Sex	Female	46 (61%)	37 (51%)	0.23
	Male	30 (39%)	36 (49%)	
ECOG performance status	0	34 (45%)	20 (27%)	0.062
	1	34 (45%)	39 (53%)	
	2	8 (10%)	14 (19%)	
Disease stage according to TNM v7	I	3 (4%)	3 (4%)	0.91
	II	10 (13%)	11 (15%)	
	III	63 (83%)	58 (80%)	
	Missing	0	1 (1%)	
T descriptor	T1	17 (22%)	13 (18%)	0.028
	T2	20 (26%)	6 (8%)	
	T3	11 (15%)	14 (19%)	
	T4	24 (32%)	32 (44%)	
	Missing	4 (5%)	8 (11%)	
N descriptor	N0	10 (13%)	19 (26%)	0.022
	N1	14 (18%)	5 (7%)	
	N2	27 (36%)	23 (31%)	
	N3	23 (30%)	18 (25%)	
	Missing	2 (3%)	8 (11%)	
Pleural fluid	Present	5 (7%)	7 (10%)	0.50
Weight loss last 3 months before inclusion	≥5%	16 (21%)	23 (31%)	0.28

PET-CT, ^18^F-fluorodeoxyglucose positron emission tomography-computed tomography; SNI, selective nodal irradiation; ENI, elective nodal irradiation; ECOG, Eastern Cooperative Oncology Group.

### Treatment Completion and Response

There was no significant difference in mean number of chemotherapy courses (PET-CT/SNI: 3.9, CT/ENI: 3.8, *p* = 0.093) or in proportions who had a dose reduction (PET-CT/SNI: 80%, CT/ENI: 67%, *p* = 0.068), but more patients in the PET-CT/SNI group received carboplatin instead of cisplatin (42% versus 4%, *p* < 0.001). There was no difference in proportions who completed TRT as planned (PET-CT/SNI: 96%, CT/ENI: 97%, *p* = 1.00). In the PET-CT/SNI group, four-dimensional CT simulation was done in 54 patients (71%) and 24 (32%) were treated with IMRT or VMAT. PTV was reported for all patients in the PET-CT/SNI group. In the CT/ENI group, PTV was available for 60 patients (82%, reported for 42 patients and estimated for 18). Median PTV was significantly smaller in the PET-CT/SNI group (320 cm^3^ [range: 42–1159] versus 760 cm^3^ [range: 189–2107], *p* < 0.001). There was no difference in proportions who received PCI (PET-CT/SNI: 84%, CT/ENI: 84%, *p* = 0.91) or second-line chemotherapy (PET-CT/SNI: 51%, CT/ENI: 43%, *p* = 0.36) (Table 2).

**Table 2:** Treatment Completion, Response to Chemoradiotherapy, and Radiotherapy-Related Toxicity

Categories	xx	PET-CT/SNI group (n=76)	CT/ENI group (n=73)	p value
Chemotherapy	Completed all 4 courses	70 (92%)	60 (82%)	0.070
	Mean number of courses (standard deviation)	3.9 (0.6)	3.8 (0.5)	0.093
	Any dose reduction	61 (80%)	49 (67%)	0.068
	Received carboplatin in ≥1 course	32 (42%)	3 (4%)	<0.001
Thoracic radiotherapy	Completed as planned	73 (96%)	71 (97%)	1.00
	Four-dimensional CT-guided target delineation	54 (71%)	-	
	IMRT or VMAT	24 (32%)	-	
	Median planning target volume, cm^3^ (range)	320 (42–1159)	760 (189–2107)	<0.001
	Missing planning target volume	0	13 (18%)	
Response to chemoradiotherapy	Overall objective response rate	62 (82%)	64 (88%)	0.30
	Complete response	17 (22%)	24 (33%)	
	Partial response	45 (59%)	40 (55%)	
	Stable disease	6 (8%)	1 (1%)	
	Progressive disease	5 (7%)	3 (4%)	
	Missing	3 (4%)	5 (7%)	
Prophylactic cranial irradiation	Received	64 (84%)	61 (84%)	0.91
Second line chemotherapy	Received	39 (51%)	32 (44%)	0.36
Esophagitis, CTCAE grade	0	29 (38%)	23 (30%)	0.39
	1-2	33 (43%)	26 (36%)	0.33
	3-4	14 (18%)	24 (33%)	0.043
	5	0	0	
Pneumonitis, CTCAE grade	0	72 (95%)	61 (84%)	0.028
	1-2	4 (5%)	9 (12%)	0.13
	3-4	0	2 (3%)	0.24
	5	0	1 (1%)	0.49

PET-CT,^18^F-fluorodeoxyglucose positron emission tomography-computed tomography; SNI, selective nodal irradiation; ENI, elective nodal irradiation; IMRT, intensity-modulated radiotherapy; VMAT, volumetric-modulated arch therapy; CTCAE, Common Terminology Criteria for Adverse Events (v4.0 in PET-CT/SNI group and v3-0 in CT/ENI group).

There was no difference in overall objective response rates between the groups (PET-CT/SNI: 82%, CT/ENI: 88%, *p* = 0.30) (Table 2).

### Radiotherapy-related Toxicity

Significantly fewer patients in the PET-CT/SNI group experienced grade 3-4 esophagitis (18% versus 33%, *p* = 0.043). Still, there was no difference in proportions who experienced grade 1-2 esophagitis (43% versus 36%, *p* = 0.33). Significantly fewer patients in the PET-CT/SNI group experienced grade >=1 pneumonitis (5% versus 16%, *p* = 0.028). Two patients experienced grade 3-4 pneumonitis and one died from pneumonitis in the CT/ENI group (Table 2).

In total, there were four treatment-related deaths (PET-CT/SNI: n = 1, CT/ENI: n = 3). The patient in the PET-CT/SNI group died from thrombocytopenic bleeding. The patients in the CT/ENI group died from pneumonitis, myocardial infarction, and respiratory failure.

### Health-related QOL

**Figure 2:**
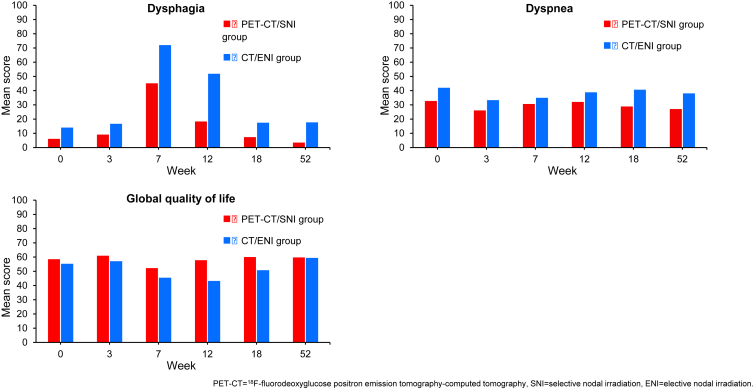
Mean scores for primary HRQOL end points. A higher score on the dysphagia and dyspnea scale indicates more symptoms, while a higher score on the global quality of life scale indicates better HRQoL. CT, computed tomography;ENI, elective nodal irradiation; HRQoL, health-related quality of life; PET, positron emission tomography; SNI, selective nodal irradiation.

Patients in the PET-CT/SNI group reported a clinically significant lower mean score of dysphagia at the end of TRT (45 versus 72). They also reported less dysphagia at weeks 12, 18, and 52, less dyspnea at weeks 18 and 52, and better global QoL at week 12. Otherwise, there were no clinically relevant differences in HRQoL scores between the groups (Fig. 2).

### OS and PFS

**Figure 3:**
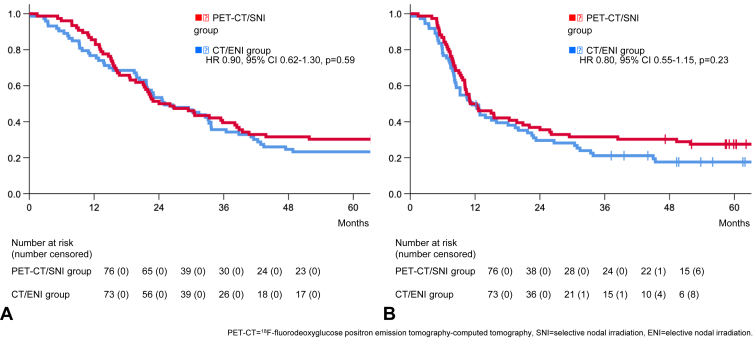
Comparison of (*A*) overall survival, and (*B*) progression-free survival. CT, computed tomography; ENI, elective nodal irradiation; PET, positron emission tomography; SNI, selective nodal irradiation.

There was no difference in median OS (PET-CT/SNI: 24 mo [95% confidence interval (CI): 15–33], CT/ENI: 25 mo [95% CI: 17–33], hazard ratio [HR] = 0.90 [95% CI: 0.62–1.30], *p* = 0.59) (Fig. 3*A*) or in median PFS (PET-CT/SNI: 11 mo [95% CI: 6–16], CT/ENI: 11 mo [95% CI: 8–15], HR = 0.80 [95% CI: 0.55–1.15] *p* = 0.23) (Fig. 3*B*). At five years, 23 patients (30%, 95% CI: 20–42) in the PET-CT/SNI group were alive, compared with 17 patients (23%, 95% CI: 14–35) in the CT/ENI group (odds ratio = 1.43, 95% CI: 0.69–2.97), *p* = 0.34).

In multivariable analyses, there was no significant difference in OS (PET/CT/SNI versus CT/ENI; HR = 0.93, 95% CI: 0.63–1.38, *p* = 0.73) or in 5-year OS (odds ratio = 0.73, 95% CI: 0.33–1.63, *p* = 0.44). Female sex was an independent positive prognostic factor for OS (HR = 0.64, 95% CI: 0.44–0.94, *p* = 0.024), while higher age (HR = 1.03, 95% CI: 1.01–1.06, *p* = 0.010), poor performance status (2 versus 0; HR = 2.06, 95% CI: 1.18–3.61, *p* = 0.011), and stage III disease (stage III versus I–II; HR = 1.88, 95% CI: 1.13–3.13, *p* = 0.016) were independent negative prognostic factors. None of these factors were significantly associated with 5-year OS (Table 3).

**Table 3:** Multivariable Analyses of Overall Survival and 5-year Overall Survival

xx	xx	xx	Overall survival		5-year overall survival	
		Number of cases	Hazard ratio (95% CI)	p value	Odds ratio (95% CI)	p value
Study group	CT/ENI	72	1 (ref)	-	1 (ref)	-
	PET-CT/SNI	76	0.93 (0.63-1.38)	0.73	0.73 (0.33-1.63)	0.44
Age	Per year	148	1.03 (1.01–1.06)	0.010	0.95 (0.91–1.00)	0.051
Sex	Male	65	1 (ref)	-	1 (ref)	-
	Female	83	0.64 (0.44–0.94)	0.024	1.90 (0.85–4.26)	0.12
ECOG performance status	0	54	1 (ref)	-	1 (ref)	-
	1	72	1.52 (0.99–2.34)	0.054	0.72 (0.32–1.62)	0.43
	2	22	2.06 (1.18–3.61)	0.011	0.20 (0.04–1.01)	0.051
Disease stage	I-II	27	1 (ref)	-	1 (ref)	-
	III	121	1.88 (1.13–3.13)	0.016	0.52 (0.20–1.35)	0.18

PET-CT,^18^F-fluorodeoxyglucose positron emission tomography-computed tomography; SNI, selective nodal irradiation; ENI, elective nodal irradiation; ECOG, eastern cooperative oncology group.

## Discussion

In this study comparing LS SCLC patients who received TRT of 45 Gy/30 fractions in two randomized trials, we found no significant difference in OS or PFS between patients who had a PET-CT for staging and received SNI and patients who were staged using CT and received ENI. However, patients in the PET-CT/SNI group experienced significantly less radiotoxicity and reported less dysphagia after TRT, probably because the PTVs in this group were significantly smaller than in the CT/ENI group.

The main effect of PET-CT on survival is believed to be that SCLC patients who truly have LS are better identified than when only using CT for staging. Consequently, patients with LS according to PET-CT should have a better prognosis and possibly be the ones who benefit the most from chemoradiotherapy. We are, however, only aware of three previous studies comparing survival between patients staged with and without PET or PET-CT in LS SCLC.^27^, ^28^, ^29^ In a subgroup analysis of the CONVERT trial (n = 540), there was no significant difference in survival between patients staged with and without PET-CT, though those staged with PET-CT had eight months longer median OS (31 versus 23 mo, *p* = 0.19) and three months longer median PFS (17 versus 14 mo, *p* = 0.20).^29^ In a small (n = 54), retrospective, single-institution study, the difference was larger and significant in favor of those staged with PET (n = 30) (median OS 32 versus 17 mo, *p* = 0.03).^28^ Another retrospective study extracted data from the Veterans Affairs Central Cancer Registry (VACCR) on LS patients who received concurrent chemoradiotherapy between 2001 and 2010 in the United States (n = 1536) and found significantly longer survival among those staged with PET (n = 397) (median OS 20 versus 14 mo, *p* < 0.001). In contrast, there was no survival benefit in our study, but the studies are not necessarily directly comparable. We used a more liberal definition of LS than in CONVERT (did not allow spread to contralateral hilar or supraclavicular region),^29^^,^^32^ which might have led to fewer patients being upstaged. PET-CT and brain MRI were mandatory for all patients with tentative LS after CT staging in our THORA trial. This was not the case in the three previous studies, and the use of PET-CT might not have been completely random: In CONVERT, more patients staged with PET-CT received 6 chemotherapy courses (25% versus 16%, *p* = 0.026),^29^ and PET-CT staged patients in the VACCR study were more likely to undergo a brain MRI at baseline (42% versus 20%, *p* < 0.001).^27^ Another important difference is that none of the patients in CONVERT or the single-institution study received ENI (data on target volume definitions was not reported in the VACCR study).^28^^,^^29^ The isolated nodal failure rate is higher after SNI based on CT than after SNI based on PET-CT (<11% versus <3%),^15^, ^16^, ^17^^,^^39^, ^40^, ^41^, ^42^, ^43^ and in a small retrospective study by Han et al.,^17^ survival was inferior among those who received SNI after CT alone (n = 30) (3-year survival: SNI: 29%, ENI: 56%, *p* = 0.022), but not among those who had a PET-CT (n = 50) (3-year survival: SNI: 53%, ENI: 52%, *p* = 0.96).^17^ The latter is supported by another small retrospective study by Suzuki et al.^19^ (n = 37) (2-year OS: 47% versus 62%, *p* = 0.77).

To our knowledge, the two retrospective studies are the only previous studies comparing toxicity between SNI and ENI in LS SCLC.^17^^,^^19^ Han et al.^17^ found similar frequencies of grade ≥3 esophagitis and pneumonitis (SNI: 10% versus ENI: 13% for both toxicities, *p* = 0.77), while Suzuki et al.^19^ found significantly less grade ≥2 esophagitis after SNI (33% versus 68%, *p* = 0.014). In the CONVERT trial, there was no significant difference in acute toxicity between patients staged with and without PET-CT (grade ≥3 esophagitis: 16% versus 20%, Grade >=1 pneumonitis: 6% versus 8%), probably since all participants in that trial received SNI.^29^ Interestingly, patients staged with PET-CT experienced significantly less late esophagitis, had a significantly smaller gross tumor volume, and received lower doses to organs at risk,^29^ possibly due to a more precise definition of lesions and the better ability of PET-CT to distinguish tumors from atelectasis.^44^^,^^45^ The frequencies of grade ≥3 esophagitis (16% versus 18%) and grade ≥1 pneumonitis (6% versus. 5%) in the PET-CT/SNI groups are comparable in CONVERT and our study and are also at the same level as in other trials allowing PET-CT and omitting ENI (grade ≥3 esophagitis: 16%–19% after 45 Gy twice-daily).^6^^,^^7^^,^^46^

There are probably also other reasons than the omission of ENI for the relatively low frequency of severe radiotoxicity in our PET-CT/SNI group. IMRT and VMAT improve conformity of radiotherapy fields and reduce doses to normal tissue, and studies suggest that these techniques are associated with lower toxicity than 3D CRT.^47^^,^^48^ Use of IMRT and VMAT was limited (32% in the PET-CT/SNI group), but also 3D CRT has improved during the study period, and it is difficult to assess the impact without comparing radiotherapy plans more in detail, which was beyond the scope of this study. Furthermore, the stricter eligibility criteria (especially pulmonary function) and protocol recommendations for normal tissue irradiation in the PET-CT/SNI group might be reasons for less toxicity (Supplementary Tables 1 and 2).

Notably, we introduced PET-CT for both staging and target volume definition in the THORA trial, which makes it difficult to accurately assess the effect of each measure. Study limitations include the sample size, the differences in eligibility criteria and normal tissue constraints, and the lack of data on relapse patterns (unavailable in the CT/ENI group). A detailed review of relapse patterns among participants in the THORA trial will be published later. The THORA trial was not designed to collect outcome data on patients who were upstaged from LS to extensive stage based on PET-CT findings. There have been concerns about using PET-CT for treatment selection in this setting,^29^^,^^49^ since it cannot be ruled out that some patients who are upstaged by PET-CT may also benefit from being treated as having LS.^50^

We are not aware of any prospective randomized trial comparing outcomes of ENI and SNI in LS SCLC, but ENI has been omitted in most recent trials of TRT in LS SCLC.^6^^,^^7^^,^^31^^,^^46^ Results of our study explain why omitting ENI reduces radiotoxicity and supports the use of PET-CT for staging and target volume definition in LS SCLC. The combination with modern radiotherapy techniques causes much less toxicity than in the Intergroup 0096 trial,^20^ and should facilitate the use of (twice-daily) TRT, particularly higher doses including the 60 Gy twice-daily schedule which was well tolerated and led to significantly improved survival in our THORA trial.^31^ There was no significant benefit in terms of OS or PFS, but our data support other evidence showing that SNI based on PET-CT provides at least as good disease control as ENI.^15^, ^16^, ^17^, ^18^, ^19^ After all, median OS and 5-year survival in recent trials omitting ENI is still better than in the Intergroup 0096 trial, and it has been shown that PET-CT-based SNI sometimes ensures irradiation of lesions missed when applying ENI.^15^^,^^51^ On the other hand, one might have expected that using PET-CT would exclude some patients with more widespread disease than detected on CT alone and thereby improve survival. A possible explanation for not detecting such a survival benefit in our study is that applying ENI leads to the irradiation of micro-metastases not detectable on PET-CT. Our ongoing study of relapse locations will provide more information on the potential limitations of applying SNI.

In conclusion, compared with CT staging and ENI, we found that using PET-CT for staging and target volume definition in LS SCLC led to a significant and clinically relevant reduction in acute radiotoxicity and patient-reported symptoms without compromising disease control or survival.

## CRediT Authorship Contribution Statement

**Gustav Graabak:** Formal analysis, Writing - original draft, Writing - review and editing, Visualization.

**Bjørn Henning Grønberg:** Conceptualization, Methodology, Writing - review and editing, Supervision.

**Kristin Toftaker Killingberg:** Methodology, Writing - review and editing, Supervision.

**Tarje Onsøien Halvorsen:** Conceptualization, Methodology, Writing - original draft, Writing - review and editing, Supervision.

## Disclosure

The authors declare no conflict of interest.

## References

[1] Govindan R., Page N., Morgensztern D. (2006). Changing epidemiology of small-cell lung cancer in the United States over the last 30 years: analysis of the surveillance, epidemiologic, and end results database. J Clin Oncol.

[2] Cancer Registry of Norway Lung cancer annual report 2022. https://www.kreftregisteret.no/Generelt/Rapporter/Arsrapport-fra-kvalitetsregistrene/Arsrapport-for-lungekreft/.

[3] Sundstrøm S., Bremnes R.M., Kaasa S. (2002). Cisplatin and etoposide regimen is superior to cyclophosphamide, epirubicin, and vincristine regimen in small-cell lung cancer: results from a randomized phase III trial with 5 years’ follow-up. J Clin Oncol.

[4] Warde P., Payne D. (1992). Does thoracic irradiation improve survival and local control in limited-stage small-cell carcinoma of the lung? A meta-analysis. J Clin Oncol.

[5] Pignon J.P., Arriagada R., Ihde D.C. (1992). A meta-analysis of thoracic radiotherapy for small-cell lung cancer. N Engl J Med.

[6] Faivre-Finn C., Snee M., Ashcroft L. (2017). Concurrent once-daily versus twice-daily chemoradiotherapy in patients with limited-stage small-cell lung cancer (CONVERT): an open-label, phase 3, randomised, superiority trial. Lancet Oncol.

[7] Bogart J., Wang X., Masters G. (2023). High-dose once-daily thoracic radiotherapy in limited-stage small-cell lung cancer: CALGB 30610 (Alliance)/RTOG 0538. J Clin Oncol.

[8] Gronberg B.H.H., Killingberg K.T., Fløtten Ø. (2023). Final survival data from a randomized phase II trial comparing high dose with standard-dose twice-daily (BID) thoracic radiotherapy (TRT) in limited stage small-cell lung cancer (LS SCLC). J Clin Oncol.

[9] Martucci F., Pascale M., Valli M.C. (2020). Impact of ^18^F-FDG PET/CT in staging patients with small cell lung cancer: a systematic review and meta-analysis. Front Med (Lausanne).

[10] Kalemkerian G.P., Gadgeel S.M. (2013). Modern staging of small cell lung cancer. J Natl Compr Canc Netw.

[11] Thomson D., Hulse P., Lorigan P., Faivre-Finn C. (2011). The role of positron emission tomography in management of small cell lung cancer. Lung Cancer.

[12] Ruben J.D., Ball D.L. (2012). The efficacy of PET staging for small-cell lung cancer: a systematic review and cost analysis in the Australian setting. J Thorac Oncol.

[13] Mitchell M.D., Aggarwal C., Tsou A.Y., Torigian D.A., Treadwell J.R. (2016). Imaging for the pretreatment staging of small cell lung cancer: a systematic review. Acad Radiol.

[14] Ambrosini V., Nicolini S., Caroli P. (2012). PET/CT imaging in different types of lung cancer: an overview. Eur J Radiol.

[15] van Loon J., De Ruysscher D., Wanders R. (2010). Selective nodal irradiation on basis of (18)FDG-PET scans in limited-disease small-cell lung cancer: a prospective study. Int J Radiat Oncol Biol Phys.

[16] Shirvani S.M., Komaki R., Heymach J.V., Fossella F.V., Chang J.Y. (2012). Positron emission tomography/computed tomography-guided intensity-modulated radiotherapy for limited-stage small-cell lung cancer. Int J Radiat Oncol Biol Phys.

[17] Han T.J., Kim H.J., Wu H.G., Heo D.S., Kim Y.W., Lee S.H. (2012). Comparison of treatment outcomes between involved-field and elective nodal irradiation in limited-stage small cell lung cancer. Jpn J Clin Oncol.

[18] Bütof R., Gumina C., Valentini C. (2017). Sites of recurrent disease and prognostic factors in SCLC patients treated with radiochemotherapy. Clin Transl Radiat Oncol.

[19] Suzuki G., Yamazaki H., Aibe N. (2022). ＜Editors' Choice＞ Elective nodal irradiation versus involved field radiotherapy for limited disease small cell lung cancer: a single-institution experience. Nagoya J Med Sci.

[20] Turrisi A.T., Kim K., Blum R. (1999). Twice-daily compared with once-daily thoracic radiotherapy in limited small-cell lung cancer treated concurrently with cisplatin and etoposide. N Engl J Med.

[21] Farrell M.J., Yahya J.B., Degnin C. (2019). Radiation dose and fractionation for limited-stage small-cell lung cancer: survey of US radiation oncologists on practice patterns. Clin Lung Cancer.

[22] Norwegian Lung Cancer Group National guidelines for diagnosis, treatment and follow-up of lung cancer, mesothelioma and thymoma. https://www.helsedirektoratet.no/retningslinjer/lungekreft-mesoteliom-og-thymom-handlingsprogram.

[23] National Comprehensive Cancer Network (2024). NCCN clinical practice guidelines in oncology: small cell lung cancer v1. https://www.nccn.org/professionals/physician_gls/pdf/sclc.pdf.

[24] Dingemans A.C., Früh M., Ardizzoni A. (2021). Small-cell lung cancer: ESMO Clinical Practice Guidelines for diagnosis, treatment and follow-up^☆^. Ann Oncol.

[25] National Institute for Health and Care Excellence Lung cancer: diagnosis and management. https://www.nice.org.uk/guidance/ng122.

[26] Daly M.E., Ismaila N., Decker R.H. (2021). Radiation therapy for small-cell lung cancer: ASCO guideline endorsement of an ASTRO guideline. J Clin Oncol.

[27] Hong J.C., Boyer M.J., Spiegel D.Y. (2019). Increasing PET use in small cell lung cancer: survival improvement and stage migration in the VA central cancer registry. J Natl Compr Canc Netw.

[28] Xanthopoulos E.P., Corradetti M.N., Mitra N. (2013). Impact of PET staging in limited-stage small-cell lung cancer. J Thorac Oncol.

[29] Manoharan P., Salem A., Mistry H. (2019). ^18^F-Fludeoxyglucose PET/CT in SCLC: analysis of the CONVERT randomized controlled trial. J Thorac Oncol.

[30] Grønberg B.H., Halvorsen T.O., Fløtten Ø. (2016). Randomized phase II trial comparing twice daily hyperfractionated with once daily hypofractionated thoracic radiotherapy in limited disease small cell lung cancer. Acta Oncol.

[31] Grønberg B.H., Killingberg K.T., Fløtten Ø. (2021). High-dose versus standard-dose twice-daily thoracic radiotherapy for patients with limited stage small-cell lung cancer: an open-label, randomised, phase 2 trial. Lancet Oncol.

[32] Stahel R.A., Ginsberg R., Havemann K. (1989). Staging and prognostic factors in small cell lung cancer: a consensus report. Lung Cancer.

[33] Shepherd F.A., Crowley J., Houtte P.V. (2007). The international association for the study of lung cancer staging project: proposals regarding the clinical staging of small cell lung cancer in the forthcoming seventh edition of the tumor, node, metastasis classification for lung cancer. J Thorac Oncol.

[34] Therasse P., Arbuck S.G., Eisenhauer E.A. (2000). New guidelines to evaluate the response to treatment in solid tumors. European Organization for Research and Treatment of Cancer, National Cancer Institute of the United States, National Cancer Institute of Canada. J Natl Cancer Inst.

[35] Eisenhauer E.A., Therasse P., Bogaerts J. (2009). New response evaluation criteria in solid tumours: revised RECIST guideline (version 1.1). Eur J Cancer.

[36] Trotti A., Colevas A.D., Setser A. (2003). CTCAE v3.0: development of a comprehensive grading system for the adverse effects of cancer treatment. Semin Radiat Oncol.

[37] Fayers P., Aaronson N.K., Bjordal K., Groenvold M., Curran D., Bottomley A. (2001).

[38] Maringwa J.T., Quinten C., King M. (2011). Minimal important differences for interpreting health-related quality of life scores from the EORTC QLQ-C30 in lung cancer patients participating in randomized controlled trials. Support Care Cancer.

[39] De Ruysscher D., Bremer R.H., Koppe F. (2006). Omission of elective node irradiation on basis of CT-scans in patients with limited disease small cell lung cancer: a phase II trial. Radiother Oncol.

[40] Xia B., Chen G.Y., Cai X.W. (2012). Is involved-field radiotherapy based on CT safe for patients with limited-stage small-cell lung cancer?. Radiother Oncol.

[41] Colaco R., Sheikh H., Lorigan P. (2012). Omitting elective nodal irradiation during thoracic irradiation in limited-stage small cell lung cancer--evidence from a phase II trial. Lung Cancer.

[42] Baas P., Belderbos J.S., Senan S. (2006). Concurrent chemotherapy (carboplatin, paclitaxel, etoposide) and involved-field radiotherapy in limited stage small cell lung cancer: a Dutch multicenter phase II study. Br J Cancer.

[43] Watkins J.M., Wahlquist A.E., Zauls A.J. (2010). Involved-field radiotherapy with concurrent chemotherapy for limited-stage small-cell lung cancer: disease control, patterns of failure and survival. J Med Imaging Radiat Oncol.

[44] Nestle U., Walter K., Schmidt S. (1999). 18F-deoxyglucose positron emission tomography (FDG-PET) for the planning of radiotherapy in lung cancer: high impact in patients with atelectasis. Int J Radiat Oncol Biol Phys.

[45] Yin L.J., Yu X.B., Ren Y.G., Gu G.H., Ding T.G., Lu Z. (2013). Utilization of PET-CT in target volume delineation for three-dimensional conformal radiotherapy in patients with non-small cell lung cancer and atelectasis. Multidiscip Respir Med.

[46] Qiu B., Li Q., Liu J. (2021). Moderately hypofractionated once-daily compared with twice-daily thoracic radiation therapy concurrently with etoposide and cisplatin in limited-stage small cell lung cancer: a multicenter, Phase II, randomized trial. Int J Radiat Oncol Biol Phys.

[47] Shirvani S.M., Juloori A., Allen P.K. (2013). Comparison of 2 common radiation therapy techniques for definitive treatment of small cell lung cancer. Int J Radiat Oncol Biol Phys.

[48] Chun S.G., Hu C., Choy H. (2017). Impact of intensity-modulated radiation therapy technique for locally advanced non-small-cell lung cancer: a secondary analysis of the NRG oncology RTOG 0617 randomized clinical trial. J Clin Oncol.

[49] Manoharan P., Salem A., Mistry H., Faivre-Finn C. (2019). Letter to the editor: increasing PET use in small cell lung cancer. J Natl Compr Canc Netw.

[50] Slotman B.J., van Tinteren H., Praag J.O. (2015). Use of thoracic radiotherapy for extensive stage small-cell lung cancer: a phase 3 randomised controlled trial. Lancet.

[51] Niho S., Fujii H., Murakami K. (2007). Detection of unsuspected distant metastases and/or regional nodes by FDG-PET [corrected] scan in apparent limited-disease small-cell lung cancer. Lung Cancer.

