# Supplementary material for: Effect of FDG PET-CT for Staging and Radiotherapy Planning – A Comparison of Cohorts From Two Randomized Trials of Thoracic Radiotherapy in Limited-Stage SCLC
Source: JTO Clin Res Rep. 2024 May 16;5(9):100688. doi: 10.1016/j.jtocrr.2024.100688 (PMC11404135; doi:10.1016/j.jtocrr.2024.100688)
Supplement: Supplementary Tables 1-2 [file mmc1.pdf]

Supplementary table 1. Eligibility criteria.

Eligibility criteria	PET-CT/SNI group (THORA trial)	CT/ENI group (HAST trial)
Age	≥18 years old.	≥18 years old.
Diagnosis	Histologically or cytologically confirmed SCLC.	Histologically or cytologically confirmed SCLC.
Operable	Allowed if patient declined surgery.	Allowed if patient declined surgery.
Definition of limited stage	Disease confined to one hemithorax, the mediastinum, contralateral hilus, and supraclavicular regions. Pleural effusion allowed.	Disease confined to one hemithorax, the mediastinum, contralateral hilus, and supraclavicular regions. Pleural effusion allowed.
Eastern Cooperative Oncology Group performance status	0-2.	0-2.
Measurable disease at baseline and response evaluation*	Response Evaluation Criteria in Solid Tumors v1.1.	Response Evaluation Criteria in Solid Tumors v1.0.
Pulmonary function*	- Forced expiratory volume >1 L or >30% of predicted value.<br>- Diffusion capacity for carbon monoxide >30% of predicted value.	Not specified.
Other organ function*	- Absolute neutrophil count ≥1.5 x 10^9/L.<br>- Platelets ≥100 x 10^9/L.<br>- Alanine aminotransferase ≤3 x ULN.<br>- Bilirubin ≤1.5 x ULN.<br>- Creatinine <100 μmol/L.<br>- Creatinine clearance >50 mL/min.	- Leukocytes ≥3.0 x 10^9/L.<br>- Platelets ≥100 x 10^9/L.<br>- Bilirubin ≤1.5 x ULN.<br>- Creatinine <125 μmol/L.
Pleural fluid	Allowed if one cytology examination was negative.	Allowed if one cytology examination was negative.
Previous thoracic radiotherapy	Not allowed.	Not allowed.
Previous systemic treatment of SCLC*	Not allowed.	Not specified.
Other active cancer than SCLC	Not allowed.	Not allowed.
Pregnancy or lactating women	Not allowed.	Not allowed.
Serious concomitant disorders*	No serious comorbidity compromising the ability to complete study procedures, treatment, or follow-up.	No serious kidney disease.

*Different between PET-CT/SNI group and CT/ENI group. PET-CT=^{18}F-fluorodeoxyglucose positron emission tomography-computed tomography, SNI=selective nodal irradiation, ENI=elective nodal irradiation, SCLC=small-cell lung cancer.

Supplementary table 2. Radiotherapy procedures.

Procedures		PET-CT/SNI group (THORA trial)	CT/ENI group (HAST trial)
TRT	RT Technique*	3D conformal RT, intensity-modulated RT, or volumetric-modulated arch therapy.	3D conformal RT.
	Schedule	45 Gy/30 fractions (twice-daily) or 60 Gy/40 fractions (twice-daily).<br>Minimum 6 hours between fractions.	45 Gy/30 fractions (twice-daily) or 42 Gy/15 fractions (once-daily).<br>Minimum 6 hours between fractions.
	Start of TRT	21-28 days after day 1 of first chemotherapy course.	21-28 days after day 1 of first chemotherapy course.
	Targets of TRT*	Primary lung tumor plus PET-CT positive lesions (selective nodal irradiation).	All pathological lesions on baseline CT scan plus elective nodal irradiation of lymph node stations 4-7 bilaterally.
	GTV*	Primary lung tumor (GTV tumor) + avid lymph stations (GTV lymph nodes) on baseline PET-CT.	Primary lung tumor (GTV tumor) + enlarged lymph stations (GTV lymph nodes) on baseline CT.
	GTV delineation	According to size on planning CT scan after first chemotherapy course.	According to size on planning CT scan one week prior to TRT.
	CTV*	CTV(tumor) and CTV(lymph nodes): Corresponding GTV + 0.5 cm margin in all directions, although not into bony structures, large vessels, the heart, or beyond mediastinal parietal pleura (unless sign of invasion).	-CTV(tumor); GTV(tumor) + 1 cm margin in all directions.<br>-CTV(mediastinum); lymph node stations 4-7 (If GTV lymph nodes <2 cm from ENI field → extension of field to 2 cm beyond nodes, although normally not into mediastinal pleura parietalis).
	ITV*	If available, 4D-CT to define ITV margin according to local routines. If unavailable:<br>-ITV(tumor); CTV + 0.8 cm IM lateral/anterior/posterior direction and 1cm IM superiorly/inferiorly.<br>ITV(lymph nodes); CTV + 0.5 cm IM in all directions.	-ITV(tumor); CTV + 0.5-1 cm IM lateral/anterior/posterior (transverse plan) and 1.0-1.5 IM cranio-caudal.<br>-ITV(mediastinum); CTV + 0.5 cm IM all directions.
	Planning target volume	ITV + setup margin according to local routines.	ITV + setup margin according to local routines.
	Organs at risk*	-Both lung, heart, esophagus from below larynx to gastro-esophageal junction, and spinal canal delineated as organs at risk.<br>-Mean lung dose (volume of both lungs together minus GTV(tumor)) was not to exceed 20 Gy. V20 Gy(lung)<35% (preferably) and V5 Gy(lung)<65% (preferably).<br>-Spinal canal: maximum dose 54 Gy.<br>-The mean heart dose should preferably not exceed 35 Gy and was not to exceed 46 Gy. Preferably V40 Gy(heart)<80%, V45 Gy(heart)<60%, and V60 Gy(heart)<30%.<br>-Esophagus: Maximum dose 60 Gy acceptable but should preferably be lower. Mean dose should preferably not exceed 34 Gy.<br>-Brachial Plexus: Maximum dose of 60 Gy acceptable but should preferably be lower.	- Doses to normal lung tissue: Less than 50% should receive more than 20 Gy - V20 Gy(lung)<50%.<br>-Other normal tissue constraints: According to local routines.
PCI	Eligibility criterion*	Complete response, partial response, or stable disease to chemoradiotherapy.	Complete or near complete response to chemoradiotherapy.
	Schedule*	30 Gy/15 fractions or 25 Gy/10 fractions.	30 Gy/15 fractions.
	Start of PCI	Within 6 weeks after day 1 of the fourth chemotherapy course.	Within 6 weeks after day 1 of the start of fourth chemotherapy course.

*Different between PET/CT/SNI group and CT/ENI group. PET-CT=^{18}F-fluorodeoxyglucose positron emission tomography-computed tomography, SNI=selective nodal irradiation, ENI=elective nodal irradiation, RT=radiotherapy, TRT=thoracic radiotherapy, PCI=prophylactic cranial irradiation, GTV=gross tumor volume, CTV=clinical target volume, ITV=internal target volume, IM=internal margin.
